# Evaluation of a Combined Text Messaging and Online Survey Protocol for Giardiasis Case Investigation — Colorado, September 2023–May 2024

**DOI:** 10.15585/mmwr.mm7426a1

**Published:** 2025-07-17

**Authors:** Ingrid Hewitson, Amanda Tran, Alayna Younger, Rachel H. Jervis

**Affiliations:** ^1^Division of Disease Control and Public Health Response, Colorado Department of Public Health and Environment; ^2^Department of Epidemiology, Colorado School of Public Health, Anschutz Medical Campus, University of Colorado, Aurora, Colorado.

**Summary**

**What is already known about this topic?**

Using online case investigation surveys to collect exposure information during an infectious disease outbreak can save public health staff members time while maintaining data quality; however, these surveys often result in low response rates.

**What is added by this report?**

Among Colorado patients with laboratory-confirmed *Giardia* infection, text message invitations sent during September 2023–May 2024 to prompt completion of an online survey resulted in rapid collection of complete case data for approximately one third of patients. Although participation levels varied among demographic groups, text message–based survey invitations saved public health staff members an estimated 25 minutes per case and 40 hours overall, compared with historical time estimates.

**What are the implications for public health practice?**

Using text message–based survey invitations to collect data enables public health practitioners to follow up with patients who have infectious diseases, despite limited public health resources. This protocol could be used as a model for collecting a wide range of public health information.

## Abstract

Rapid completion of routine patient interviews by public health practitioners is critical for disease control and prevention efforts; however, increasing numbers of cases of certain diseases, competing priorities, and limited public health resources have made interviewing patients increasingly challenging. The Colorado Department of Public Health and Environment implemented and evaluated a combined text messaging and online survey protocol, with no telephone communication, to collect information from persons with *Giardia* infections. During September 2023–May 2024, English and Spanish text messages were sent to Colorado residents with laboratory-confirmed *Giardia* infections reported to the Colorado EpiTrax surveillance system, inviting them to opt in to an online survey. Persons who opted in received a unique survey link via text message. Opt-in and survey completion rates were evaluated by demographic characteristics, data quality, timeliness, and time saved by staff members. Among 305 persons with *Giardia* who received text messages, 131 (43%) opted in, 95 (73%) of whom completed the survey, for an overall survey completion rate of 31%. The highest survey completion rates were among adults aged 35–54 years (43%), White (34%) and non-Hispanic (33%) respondents, and those who lived in urban areas (32%). The majority (69%) of respondents completed the online survey within 1 day of receipt of the initial text message. In addition, the majority (93%–100%) of respondents answered 11 selected universal questions, and 90%–96% answered potentially sensitive questions (i.e., those on sexual history). The combined text messaging and online survey protocol facilitated more rapid contact with patients and required fewer resources than telephone interviews, saving public health staff members approximately 25 minutes per case and 40 hours overall. The protocol is flexible enough to accommodate shifting priorities and could be used to collect a wide variety of public health information (e.g., for symptom monitoring, contact tracing, and collecting vaccination or health information). Efforts to increase participation might result in higher response rates and improved efficiency and could facilitate an even quicker response.

## Introduction

Public health professionals routinely contact persons infected with, or exposed to, certain diseases for interviews to collect detailed information on demographic characteristics, occupation, and recent exposures, as well as to monitor their symptoms. Information gathered through these interviews is critical for disease surveillance, outbreak detection, and response activities (*1*). Rapid contact with affected persons reduces recall bias and enables public health practitioners to provide actionable disease control and prevention interventions (*1*). With advances in technology such as culture-independent diagnostic tests (e.g., gastrointestinal panels) that have led to increasing disease case counts, health departments need ways to reduce the time associated with contacting and evaluating persons who have positive laboratory results (*2*–*5*). In anticipation of post–COVID-19 funding decreases and competing priorities (e.g., mpox and highly pathogenic avian influenza [HPAI]), the Colorado Department of Public Health and Environment (CDPHE) assessed the feasibility of implementing a telephone-call–free interview protocol. Although reporting of *Giardia* infection is required in Colorado, at the time the pilot protocol was implemented, interviews of persons with giardiasis were optional and conducted at the discretion of the investigating health jurisdiction. During the study period, 57 health jurisdictions were responsible for public health duties for 64 counties. CDPHE piloted a text messaging and online survey protocol for interviewing persons with *Giardia* infections in the 36 health jurisdictions for which they were responsible. Public health in Colorado is decentralized, with state and local health jurisdictions holding equal authority. Previously, local health jurisdictions conducted routine enteric disease case interviews with approximately 75% of persons with laboratory-confirmed *Giardia* infection*. *In 2018, *Giardia* interviews became optional in Colorado. Many jurisdictions stopped conducting interviews. In addition, during the COVID-19 pandemic, many jurisdictions could not maintain routine enteric disease interviews. CDPHE and the Colorado School of Public Health created the Enteric Disease Interview Team (EDIT) to conduct interviews for local jurisdictions that request assistance. This program continues successfully; EDIT staff conducted interviews for 36 local health jurisdictions during the study period. CDPHE now offers *Giardia* case interviews only through the online survey. To assess the text messaging and online survey, CDPHE evaluated the percentages and demographic characteristics of persons who 1) opted in to receive the survey via text message and 2) completed the survey. The evaluation included an assessment of data quality and estimation of time saved by staff members.

## Methods

### Online Survey Development

The online *Giardia* infection case investigation survey was developed using Research Electronic Data Capture (REDCap; Vanderbilt University) survey software that complies with Health Insurance Portability and Accountability Act standards. The survey included 32 questions asked of all respondents on nonsensitive topics (clinical information, contacts, travel, sources of exposure, occupation, and demographic characteristics) and potentially sensitive topics (including sexual history). Depending on responses, certain answers could lead to additional questions (such as questions about travel) and could result in up to 203 questions (*6*). A seven-question optional user satisfaction survey was offered after completion of the primary survey. Only persons with laboratory-confirmed *Giardia* infection who were assigned to CDPHE were offered this online survey; other persons with *Giardia* infection not assigned to CDPHE were either offered telephone interviews or were not contacted, at the discretion of the local health departments.

### Interview Protocol and Exclusions

During September 1, 2023–May 30, 2024, personalized English and Spanish text messages were sent to all persons with laboratory-confirmed *Giardia* infections that were reported to the Colorado EpiTrax surveillance system by laboratories and physicians and assigned to CDPHE as the investigating agency. Telephone numbers were received through electronic laboratory reporting with the test result. If a number was not available, medical and vaccination records were checked. Text message recipients were invited to opt in to the online survey by responding “yes” to the text message (Supplementary Figure). If no response was received, one follow-up message was sent after 24 hours.

Among 564 laboratory-confirmed *Giardia *cases reported during the study period, 341 were assigned to CDPHE. Among these, 20 were excluded; these included 1) cases with no laboratory results (three cases), 2) cases outside of Colorado (one case), and 3) cases involving co-infections with another pathogen (16 cases). Among the remaining cases, persons who did not have a mobile telephone number (e.g., with a landline only or no telephone at all) were also excluded (16 cases). 

Persons who opted in were sent a unique survey link using Twilio, a cloud-based text messaging platform integrated within REDCap. A CDPHE interviewer initiated the survey in REDCap, which instructed Twilio to send a text message with a unique survey link. When the link was clicked, the survey opened in the user’s browser within a REDCap window. Each survey link was unique to prevent data overlap. If the survey was not completed, an automatic reminder text message was sent every 2 days, up to 3 times. Data were collected and stored by REDCap.

Persons who did not opt in or who opted in but did not complete at least the core set of 23 of 32 universal questions (72%) were categorized as lost to follow-up. Respondents who opted in and completed 23 or more of the universal questions were categorized as having completed the survey. The first 23 questions requested information about symptoms, hospitalizations, ill contacts, travel, and occupational risks for exposure (e.g., child care, health care, and food industry). Questions related to demographic characteristics and sexual history appeared at the end of the survey, and not all respondents answered these nine questions. 

### Analysis

The opt-in and completion rates were evaluated by age, sex, race and ethnicity, and urbanicity of residence (*7*). Data quality was assessed by calculating the proportion of missing answers for closed-ended questions (i.e., those with defined answer options such as “yes” or “no”) by question type (nonsensitive or sensitive); timeliness (number of days from initial text message to online survey completion); and time savings for staff members, by estimating the time that would have been required to conduct telephone interviews with all surveyed persons. Frequencies and percentages were conducted using SAS software (version 9.4; SAS Institute). This study was determined by CDPHE to be nonresearch public health surveillance evaluation and was exempted from human subjects review.

## Results

### Opt-In and Completion Rates

During September 2023–May 2024, CDPHE sent text messages to 305 of 321 (95%) persons with laboratory-confirmed *Giardia *infection. Among these, 174 (57%) were categorized as opting out, with 166 nonresponses and eight refusals. A total of 131 (43%) of 305 opted in to the online survey; 89 (68%) did so after the first text message invitation and 42 (32%) after the second. Among these, 95 of 131 (73%) completed the survey. The median number of texts required to complete the survey was one (range = one to four). A total of 36 (27%) were lost to follow-up, for an overall survey completion rate of 31% (Figure).

**FIGURE:**
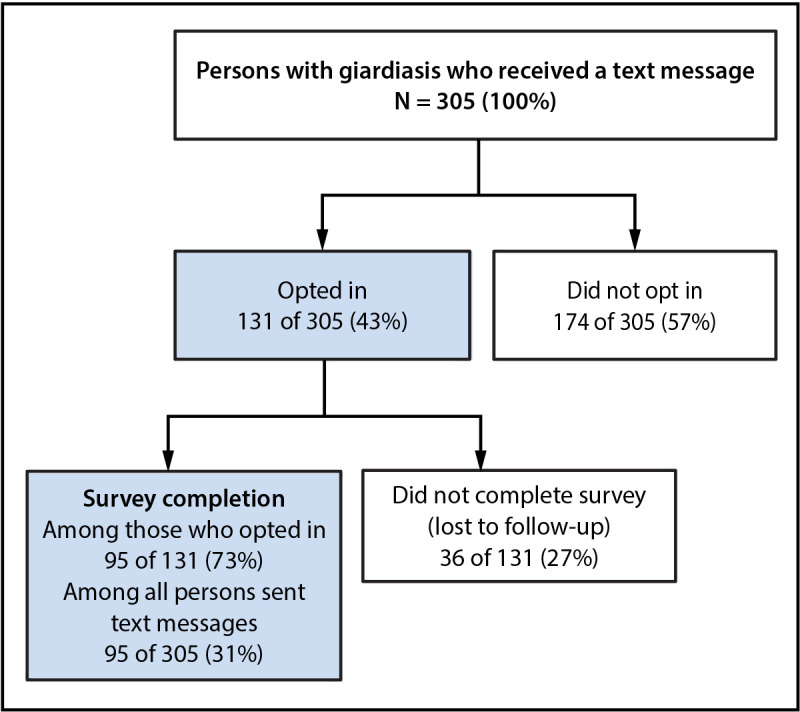
Opt-in and completion rates for online case investigation surveys sent via text message to persons with *Giardia* infection* — Colorado, September 2023–May 2024 * Persons co-infected with another pathogen, persons living outside of Colorado, persons without laboratory test results, and persons who did not have a mobile telephone number were excluded.

### Demographic Differences in Completion Rates

The survey was completed by 32% of females and 31% of males (Table 1). Among racial and ethnic groups, the highest percentages of persons who completed the survey were White (34%) or non-Hispanic (33%). Completion rates were lower among persons who were Hispanic (24%), Asian (13%), or Black or African American (14%). Among age groups, the highest percentages of completed surveys were among adults aged 45–54 years (46%) and 35–44 (40%) years, and the lowest percentages were among adults aged ≥65 years (27%), adults aged 18–24 years (24%), and parents or guardians of children aged 0–17 years (14%). Approximately one third of urban residents (32%) and one fourth of rural residents (23%) completed the survey. Among those who completed the survey, 65 (68%) also completed the user satisfaction survey. Most respondents reported that they used their mobile telephone to complete the survey (89%) and had no technical issues (97%), and none reported a desire for the survey to be in a language other than English or Spanish (100%).

**TABLE 1:** Opt-in and completion rates for online case investigation surveys sent by text message to persons with *Giardia *infection, by selected demographic characteristics — Colorado, September 2023–May 2024

Characteristic	Received text message, no. (column %)*	No. (row %)		
		Opted in	Completed survey*	Lost to follow-up*
**Total**	**305 (100)**	**131 (43)**	**95 (31)**	**210 (69)**
**Sex (n = 305)**				
Female	115 (38)	51 (44)	37 (32)	78 (68)
Male	190 (62)	80 (42)	58 (31)	132 (69)
**Race (n = 277)^†^**				
Asian	8 (3)	2 (25)	1 (13)	7 (88)
Black or African American	21 (8)	4 (19)	3 (14)	18 (86)
White	226 (82)	103 (46)	76 (34)	150 (66)
**Ethnicity (n = 288)^†^**				
Hispanic or Latino	68 (24)	32 (47)	16 (24)	52 (76)
Not Hispanic or Latino	220 (76)	92 (42)	73 (33)	147 (67)
**Age group, yrs (n = 305)**				
0–17	56 (18)	17 (30)	8 (14)	48 (86)
18–24	29 (10)	13 (45)	7 (24)	22 (76)
25–34	64 (21)	29 (45)	22 (34)	42 (66)
35–44	48 (16)	25 (52)	19 (40)	29 (60)
45–54	41 (13)	22 (54)	19 (46)	22 (54)
55–64	34 (11)	13 (38)	11 (32)	23 (68)
≥65	33 (11)	12 (36)	9 (27)	24 (73)
**Residence (n = 305)**				
Rural	39 (13)	15 (38)	9 (23)	30 (77)
Urban	266 (87)	116 (44)	86 (32)	180 (68)

* Percent is proportion of persons who received the initial text message asking them to opt in to the online survey.

^†^ Race and ethnicity data were self-reported. Persons identifying as American Indian or Alaska Native, Native Hawaiian or Pacific Islander, or other race are not included because of small sample size. Race and ethnicity percent distribution was calculated from among those for whom information was available. Persons who reported more than one race were counted in each category they selected.

### Data Quality

The majority (93%–100%) of respondents answered 11 selected universal questions. In addition, 90%–96% answered potentially sensitive questions (i.e., those on sexual history) (Table 2).

**TABLE 2:** Number and percentage of persons with *Giardia* infection who either completed all survey questions or skipped certain questions, by selected question topic and sensitivity — Colorado, September 2023–May 2024

Question topic and sensitivity	No. (%)*	
	Respondents who answered the question	Respondents who skipped the question
**Nonsensitive topic (11 questions; 95 respondents)**		
Sign or symptom		
Intermittent diarrhea	88 (93)	7 (7)
Greasy stool	92 (97)	3 (3)
Fever	92 (97)	3 (3)
Antiparasitic medication	95 (100)	0 (—)
Household characteristics		
Ill household members	95 (100)	0 (—)
Ill close contacts	94 (99)	1 (1)
Children in child care setting	94 (99)	1 (1)
Employment or volunteer position		
Residential care worker or attendee	92 (97)	3 (3)
Health care worker	95 (100)	0 (—)
Child care worker or attendee	94 (99)	1 (1)
Food handler	92 (97)	3 (3)
**Sensitive topic, sexual history (three questions; 67 respondents [aged ≥18 yrs only])**		
Sex with male partner	62 (93)	5 (7)
Sex with female partner	64 (96)	3 (4)
Sex with transgender partner	60 (90)	7 (10)

* These data reflect the number and percent of respondents who answered the question among those who were asked; they do not indicate that the person responded “yes” to the question.

### Survey Completion Time and Time Savings for Staff Members

Each online survey required approximately 5 minutes of staff member time (including reviewing patient information in EpiTrax, texting patients, and initiating REDCap survey deployment). In contrast, telephone interviews require approximately 30 minutes per interview. Staff members spent a total of approximately 8 hours administering the 95 completed online surveys, which would have required approximately 48 hours by telephone (L. Colon, Colorado Department of Public Health and Environment, personal communication, December 18, 2024). The median interval between the initial text message inviting the patient to opt in and the completion of the survey was less than 1 day (range = 0–10 days), and almost every respondent (99%) completed the survey within 6 days. Respondents completed the questions in a median of 13 minutes after opening the survey (IQR = 8–21 minutes), and 95% completed the survey within 1 hour. Illogical values were excluded, primarily those with a negative time (i.e., those in which a person appeared to have opened or completed the survey before it was recorded as having been sent, which could have been related to issues with respondents who had devices in various time zones).

## Discussion

CDPHE staff members used a combined text messaging and online survey protocol to collect data from approximately one third of patients with laboratory-confirmed giardiasis in an estimated one sixth of the time that would have been needed to complete telephone interviews (i.e., 8 hours versus an estimated 48 hours). Conducting interviews of patients with laboratory-confirmed *Giardia* became optional in 2018 because of reduced public health capacity due to increasing caseloads and limited funding and staffing. Subsequently, most Colorado agencies stopped conducting telephone interviews of patients with giardiasis. For example, during 2016–2017, a total of 75% of persons with *Giardia* infection were interviewed by telephone (E. Livesay, MPH, Colorado Department of Public Health and Environment, personal communication, April 7, 2025), whereas in 2019, only 8% were interviewed by telephone. With the combined text messaging and online survey approach described in this report, 95% of persons with laboratory-confirmed *Giardia* infection who were assigned to CDPHE were offered the survey, and 73% completed it. Because interviewing persons with *Giardia* infection is optional in Colorado (although reporting is required), these data were not likely to have been collected had the online survey not been used.

Opt-in and completion rates differed by demographic characteristics. Therefore, when evaluating the feasibility of this protocol for a specific population, pathogen, or response, understanding which populations pose the greatest public health concern (i.e., those with severe illness or at high risk for transmission) and which pathogens pose the greatest threat (i.e., those with higher transmission risk and more likely to be associated with an outbreak) is important.

The findings in this report are similar to those from other studies indicating that online surveys can be used to efficiently collect public health data. In addition, online surveys are less influenced by social desirability biases, which can occur when respondents provide the answers they think the interviewer prefers (*2*,*6*,*8*,*9*). Because most surveys were completed within 1 day of receiving the survey link, this protocol meets CDPHE’s 7-day deadline for *Giardia* infection interviews and might expedite data collection and the public health response overall. This method could also be modified for the initial data collection effort, in conjunction with telephone interviews for persons who do not opt in to the survey. Reducing the staff time needed for telephone interviews by one third would provide more time for staff members to focus on outbreak detection and address emerging infectious diseases.

Although completion rates among persons who opted in to the survey were high (73%), strategies to increase the opt-in rate should be explored. This protocol might not be appropriate for all pathogens and diseases, such as high-consequence pathogens and diseases for which immediate reporting is mandated. However, surveys have the potential to decrease the time associated with interviewing patients infected with lower-consequence pathogens such as *Giardia* and *Campylobacter *species. In addition, this protocol could be expanded for use during urgent public health situations requiring rapid or repeated contact with numerous persons, such as contact tracing or monitoring symptoms during HPAI, Ebola virus disease [Ebola], or Marburg outbreaks, to reduce the number of telephone calls required.

### Limitations

The findings in this report are subject to at least three limitations. First, because the sample size was small and limited to patients with *Giardia* infection, the findings might not be generalizable to other populations. Second, because persons who did not complete the survey were not interviewed personally, assessing systematic differences between those who did and did not respond was not possible. Finally, the giardiasis survey was relatively short, potentially resulting in higher completion rates than might have been obtained with longer surveys. 

### Implications for Public Health Practice

The combined text messaging and online survey protocol enabled public health investigators to rapidly contact persons with laboratory-confirmed *Giardia* infection using fewer resources than needed for telephone interviews. Although this approach resulted in lower data collection rates (31%) than telephone interviews during 2016–2017 (75%), and certain populations were underrepresented, the protocol allowed the health department to quickly and efficiently collect public health data from persons who would not otherwise have been interviewed (E. Livesay, MPH, Colorado Department of Public Health and Environment, personal communication, April 7, 2025). In addition, the ability for patients to provide information without participating in a personal interview might appeal to certain populations and aid in collecting sensitive information (*8*). Although this study was limited to persons with *Giardia *infection, the protocol could be adapted for use with other pathogens (e.g., *Campylobacter *species, *Bordetella* species pertussis, and West Nile virus) and to monitor symptoms for other diseases (i.e., HPAI, Ebola, and Marburg), collect vaccination information, and conduct contact tracing, particularly for pathogens with historically lower interview response rates such as *Shigella* species and *Monkeypox virus*. The protocol is flexible enough to accommodate shifting priorities and could be applied in other areas of public health to enhance the response to infectious diseases.
